# Impact of compressed sensing (CS) acceleration of two-dimensional (2D) flow sequences in clinical paediatric cardiovascular magnetic resonance (CMR)

**DOI:** 10.1007/s10334-023-01098-8

**Published:** 2023-05-18

**Authors:** Sara Moscatelli, Peter Gatehouse, Sylvia Krupickova, Raad Mohiaddin, Inga Voges, Daniel Giese, Sonia Nielles-Vallespin, Dudley J. Pennell

**Affiliations:** 1 Department of Paediatric Cardiology, Royal Brompton Hospital, Sydney Street, London, SW3 6NP UK; 2 Department of CMR, Royal Brompton Hospital, Part of Guy’s and St Thomas’ NHS Foundation Trust, Sydney Street, London, SW3 6NP UK; 3 National Heart and Lung Institute, Imperial College, London, England; 4 Department of Congenital Heart Disease and Pediatric Cardiology, University Hospital Schleswig-Holstein, Campus Kiel, Kiel, Germany; 5 Magnetic Resonance, Siemens Healthcare GmbH, Erlangen, Germany

**Keywords:** Magnetic Resonance Imaging, Blood Flow, Arteries, Pediatrics

## Abstract

**Objectives:**

Two-dimensional (2D) through-plane phase-contrast (PC) cine flow imaging assesses shunts and valve regurgitations in paediatric CMR and is considered the reference standard for Clinical quantification of blood Flow (COF). However, longer breath-holds (BH) can reduce compliance with possibly large respiratory manoeuvres altering flow. We hypothesize that reduced BH time by application of CS (Short BH quantification of Flow) (SBOF) retains accuracy while enabling faster, potentially more reliable flows. We investigate the variance between COF and SBOF cine flows.

**Methods:**

Main pulmonary artery (MPA) and sinotubular junction (STJ) planes were acquired at 1.5 T in paediatric patients by COF and SBOF.

**Results:**

21 patients (mean age 13.9, 10–17y) were enrolled. The BH times were COF mean 11.7 s (range 8.4–20.9 s) vs SBOF mean 6.5 s (min 3.6–9.1 s). The differences and 95% CI between the COF and SBOF flows were LVSV -1.43 ± 13.6(ml/beat), LVCO 0.16 ± 1.35(l/min) and RVSV 2.95 ± 12.3(ml/beat), RVCO 0.27 ± 0.96(l/min), QP/QS were SV 0.04 ± 0.19, CO 0.02 ± 0.23. Variability between COF and SBOF did not exceed intrasession variation of COF.

**Conclusion:**

SBOF reduces breath-hold duration to 56% of COF. RV flow by SBOF was biased compared to COF. The variation (95% CI) between COF and SBOF was similar to the COF intrasession test–retest 95% CI.

**Supplementary Information:**

The online version contains supplementary material available at 10.1007/s10334-023-01098-8.

## Introduction

The measurement of flows in the cardiovascular system is fundamental for understanding the physiology and hemodynamics of congenital and acquired cardiac diseases, especially in the paediatric population where these conditions are dominant [1, 2]. The analysis of flows produces important information that can guide clinical decisions, and these data are part of current cardiovascular guidelines such as the Adult Congenital Heart Diseases (ACHD) guidelines published in 2020 by the European Society of Cardiology [3]. The Cardiac Magnetic Resonance (CMR) imaging technique can acquire flow sequences from which parameters such as blood velocities, volume flow rate, total flow, and pressure gradients are obtained. These data enable us to investigate the presence not only of shunts in congenital heart defects (CHDs) but also valve regurgitations [4]. Two-dimensional (2D) through-plane phase-contrast (PC) cine flow is usually a breath-hold sequence in CMR, it is for this work considered the reference standard and is herein named Clinical quantification Of blood Flow (COF) [5–7].

Despite its “gold-standard” label, the CMR PC cine flow sequence has its own pitfalls and limitations, not least its expense, inaccessibility, slowness and requirement for patient breath-holding co-operation and tolerance of claustrophobia. Its results can be degraded by insufficient temporal or spatial resolution and modification of the true flow characteristics due to preload and afterload changes caused often by variability in respiration.

These errors can be induced by several factors such as breath-hold (BH) duration; in fact, the longer the BH, the lower can be the patient compliance with the further possibility of having large respiratory manoeuvres altering the flow characteristics [8–12] during the scanning with complex consequences. Heart rate (HR) variations during the BH, imaging parameters, and underlying comorbidities such as rhythm anomalies can have a role. Moreover, BH compliance and HR variability are even more accentuated in the paediatric population due to the physiology of these patients. In addition, scans of congenital heart diseases, where multiple flow sequences need to be performed, have a long session time in the magnet, easily causing fatigue and correspondingly reduced performance [13, 14]. However, alternative acquisition tools to reduce some of these pitfalls exist: commonly the use of free breathing with signal averaging (at an “outer loop” or longest timescale) combined with high parallel imaging factor (3 to 4) can partially overcome them with the major drawback of the tissue position being only rather approximately end-expiratory. For such free-breathing averaging, the scan time itself is not obstructively long (a common misconception) benefiting from the high parallel-acceleration, with also no need for breath-hold recovery time or time taken delivering instructions to the patient. Other technical solutions include respiratory gating, but this is less common for its extra setup time, its potential unreliability and for interruptions to the flow sequence if using diaphragmatic MRI navigators for the respiratory signal.

Another possible way to reduce these inaccuracies is to accelerate the CMR PC cine sequence acquisition to reduce the BH time and overall scan acquisition time. The application of compressed-sensing acceleration (CS), employing a pseudorandom variable density under-sampling of k-space in the spatial domain and iterative reconstruction [15] reduces the time of acquisition accepting some “fitting effect” limitations of the reconstruction. Further CS work in flow CMR is discussed later.

In our study, we measured flows in the main pulmonary artery (MPA) and sinotubular junction (STJ) planes as used in the CMR clinical practice. We hypothesize that reduced BH time by the moderate application of CS to 2D cine through-plane flows (named “Short BH quantification Of Flow”) (SBOF) retains accuracy while enabling faster and potentially more reliable paediatric flows. We, therefore, investigate the variance between COF and the new SBOF cine flows in paediatric CMR.

## Materials and methods

This observational prospective study in patients evaluating new CMR physics sequences was approved by the ethical committee of our institution (10/H0701/112–Version 4). All the data and information were always under the sole control of our institution.

### Patients

Paediatric patients with age < 18, coming to the CMR clinical service department of the Royal Brompton Hospital for routine clinical appointments, that accepted by their parents through the above informed written consent form to participate in this clinical study were enrolled between January 2021 and December 2021. All of the sequences prescribed by the protocolling consultant for the clinical investigation were acquired first, including the COF as prescribed. Only at the end of the clinical study, the CS flow sequences “SBOF” of the aortic STJ and the MPA were acquired, thereby ensuring priority of the clinical requests.

### CMR technique

All CMR examinations were performed with a 1.5 T CMR scanner (1.5 T, Avanto FIT/Aera, Siemens Healthcare GmbH). As part of the standard clinical protocol, end-expiratory breath-hold balanced steady-state free-precession (bSSFP) cines were acquired in the usual planes: left ventricular (LV) short-axis (SAX) cine, LV four-chamber, LV outflow tract, LV outflow tract cross-cut, LV two chambers, right ventricle (RV) in and out, RV two chamber, RV outflow tract and RV outflow tract cross-cut planes. The STJ and MPA conventional flow scans (COFs) were planned from the cines of the left and right ventricular outflow tracts. For aortic flow the plane was placed at the STJ, and MPA flow was acquired above the pulmonary valve. As usual for flow imaging, the velocity encoding (VENC) was optimized by the operator for each patient and plane during the COF scans (range 120–200 cm/s). The same planes and VENCs were used for the COF and compressed-sensing short breath-hold flows (SBOF) at nominally similar parameters (Table 1), using the same end-expiratory BH requests to the patient for all scans. Velocity aliasing was not accepted and flow scans were repeated at wider VENC if this occurred. In this work, a research sequence consisting of compressed-sensing 2D phase-contrast cine in k-t space was applied for SBOF with the rationale of approximately halving the BH duration of the COF sequence, at similar acquired spatial and temporal (true cine frame time) resolutions. This aim of approximately halved BH time required a moderate acceleration factor (increasing from 2 to 5, over inner to outermost ky) such that this sequence was still a “segmented k-space” acquisition over multiple cardiac cycles, as opposed to the more typical application of CS at its stronger accelerations for finer nominal resolutions or real-time cines [16]. The acceleration and reconstruction parameters (40 iterations, default regularization parameters: spatial 0.005, temporal 0.01) of the CS for the SBOF scans were held constant for all patients in the study. Gadolinium-based contrast agent was administered earlier during the clinically prescribed scans, and only if it was clinically requested. In these cases, CS sequences were acquired after the completion of late-enhancement imaging [17].

**Table 1:** Imaging parameters of the COF and SBOF methods, showing ranges because of adaptation to patients during clinical scanning

Parameter	COF	SBOF
TR (ms) at VENC 150 cm/s	4.4	4.5
TE (ms) at VENC 150 cm/s	2.3	2.3
Segments (lines per HB)	5	5
FE FOV (mm) (PE FOV adapted to plane)	320–360	320–360
FA (deg)	20	20
FE samples	224	240
Acquired resolution at 360 mm FOV (FExPE mm)	1.61 × 2.01	1.50 × 1.88
SLT (mm)	8	8
Parallel imaging (GRAPPA) factor	2	Not used
Parallel imaging coil-profile lines	24 (in scan)	Not used

### Image analysis

Analysis of the images was performed with a dedicated commercial system (cvi42 5.10, Circle Cardiovascular Imaging, Calgary, Alberta, Canada). The flow CMR images were analyzed by a single reviewer with more than three years of experience in paediatric CMR and CMR post-processing.

For flow images, contours were drawn manually during the end-diastolic phase of flow images to delineate the STJ and MPA. These contours were then propagated across all cardiac phases. Manual corrections to the ROI in each reconstructed cine frame were applied to correct for in-plane displacement and diameter and shape changes. PC background correction [25] was applied, by the 2D linear spatial fit to velocity pixels (“Static Tissue Offset”) of the Circle software, to all the flow sequences, employing stationary muscle and fat tissues distributed around the thorax wall, excluding outlying tissue far from isocentre (such as shoulders, neck or caudal abdominal regions), also excluding tissues subject to flow or motion such as the heart itself or any other blood vessels) and excluding regions of spatial phase-encoding wraparound when present. Mean blood velocity at each cardiac phase was calculated by averaging the phase-image pixel values over the vessel cross-sectional area. Volume flow was calculated by integrating the product of area and mean velocity within the contoured blood vessel. The parameters analyzed were LV stroke volumes (LVSV), RVSV, LV cardiac output (LVCO), RVCO (where CO was defined as the SV multiplied by the mean heart-rate (bpm) during each BH scan), and QP/QS (by RVSV/LVSV and also by RVCO/LVCO).

### Data analysis

Descriptive statistics of continuous quantitative measurements are summarized as means and standard deviations. The flow measurements (including the derived QP/QS) obtained by the COF and SBOF techniques were compared using paired t-test. Values of *p* < 0.05 were considered statistically significant. Stata 12 software (StataCorp LP, College Station, Texas) was used for statistical analyses.

Bland–Altman plots (17) were used to examine the differences and variabilities between the COF and SBOF measurements, which are stated as mean difference ± 2SD of the difference over all 21 patients. The significances of the mean differences were evaluated by the paired *t*-test. [18, 19].

## Results

**Fig. 1:**
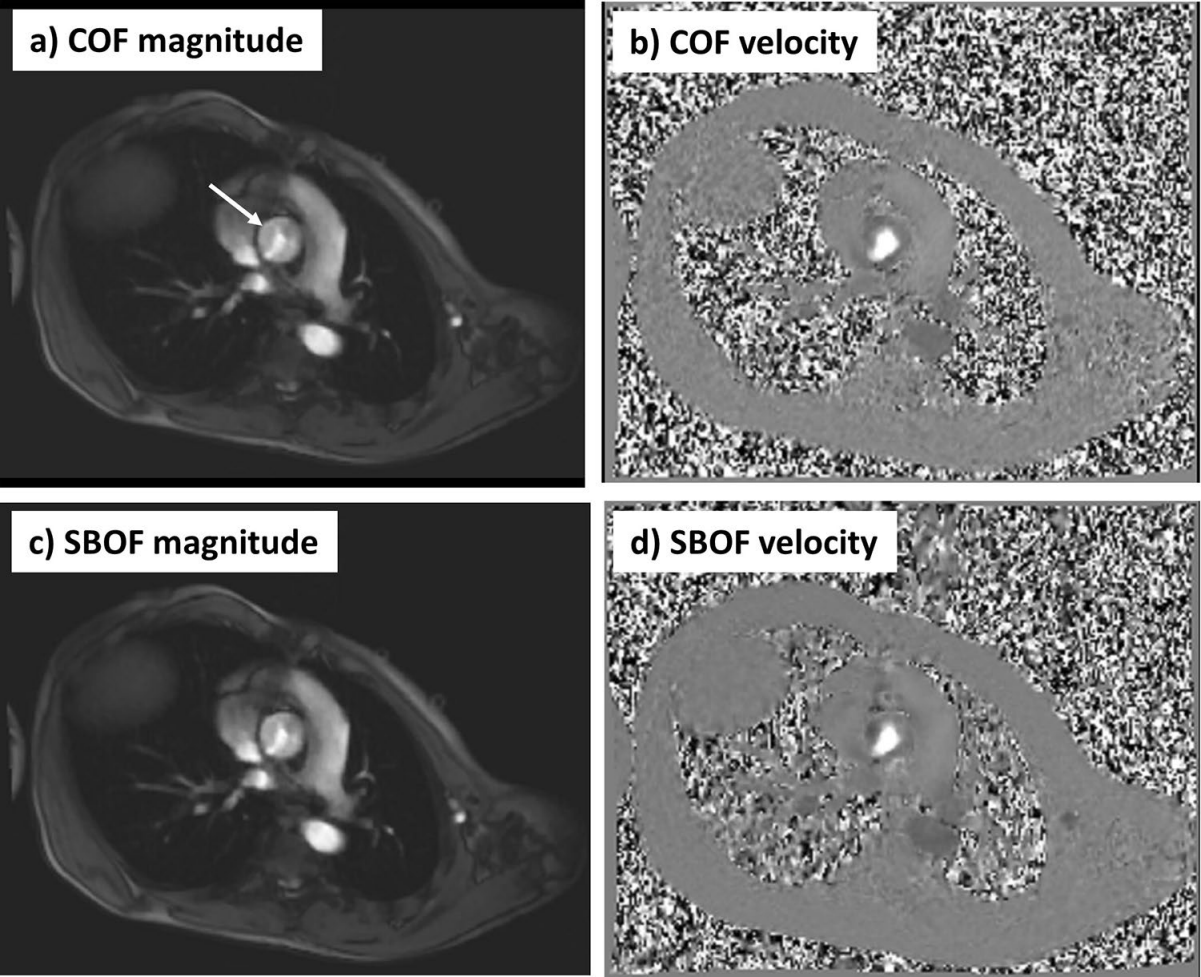
Cross-sectional imaging of the ascending aorta at the sinotubular junction (STJ, arrow) comparing the conventional flow (COF) and CS short-breath-hold flow (SBOF) in the same patient and closely matched peak-systolic cardiac timing for COF and SBOF (given variations in heart-rate between the two scans affecting the retrospectively-gated reconstructed image timings). This patient had an aortic valve stenosis (VENC = 400 cm/s). **a** COF magnitude, **b** COF velocity, **c** SBOF magnitude, **d** SBOF velocity

**Fig. 2:**
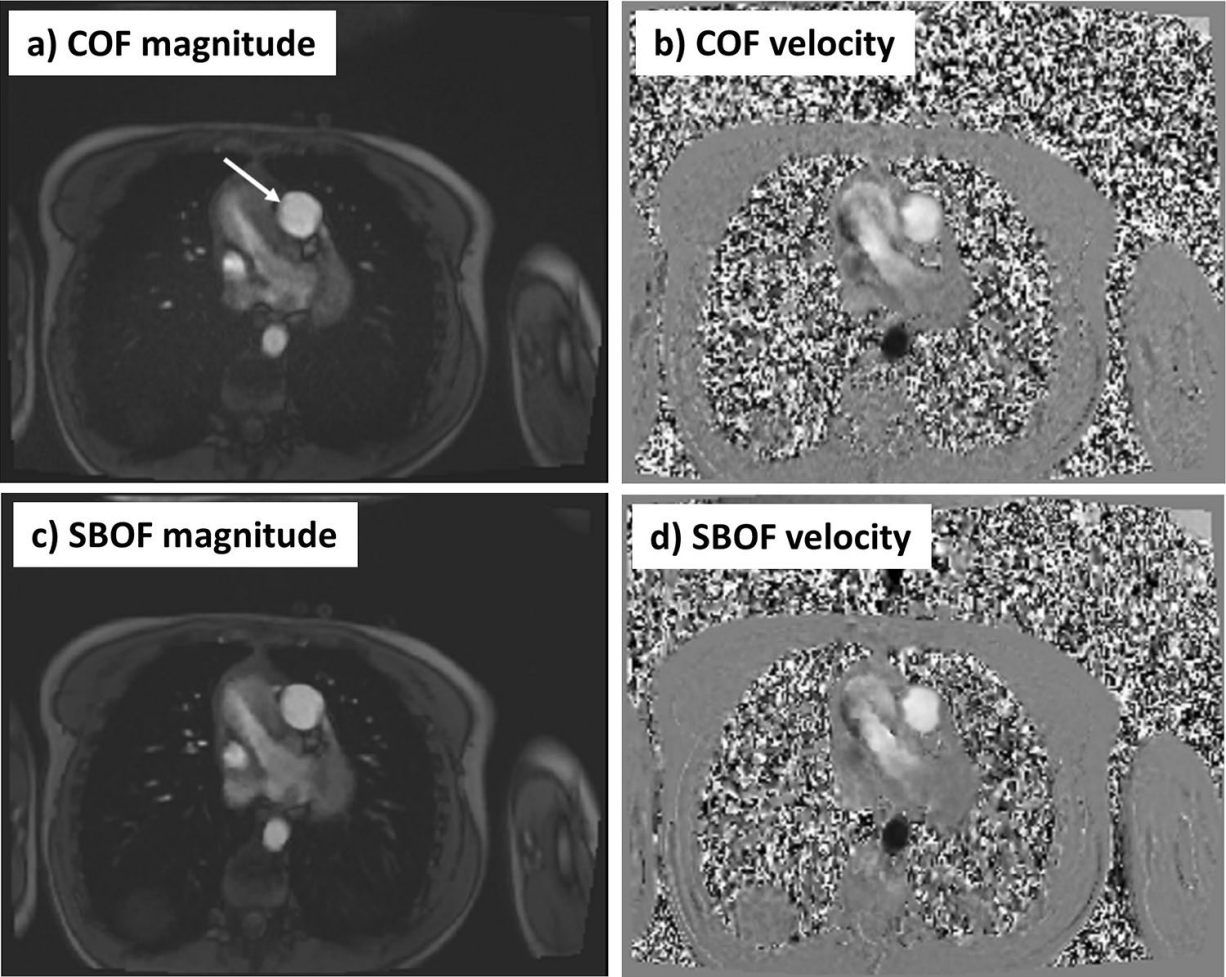
Cross-sectional imaging of the main pulmonary artery (arrow) comparing the conventional flow (COF) and CS short-breath-hold flow (SBOF) in the same patient and closely matched peak-systolic cardiac timing for COF and SBOF (given variations in heart-rate between the two scans affecting the retrospectively gated reconstructed image timings). This is the same patient as in Fig. 1, and had normal MPA flow (VENC = 150 cm/s). **a** COF magnitude, **b** COF velocity, **c** SBOF magnitude, **d** SBOF velocity

**Fig. 3:**
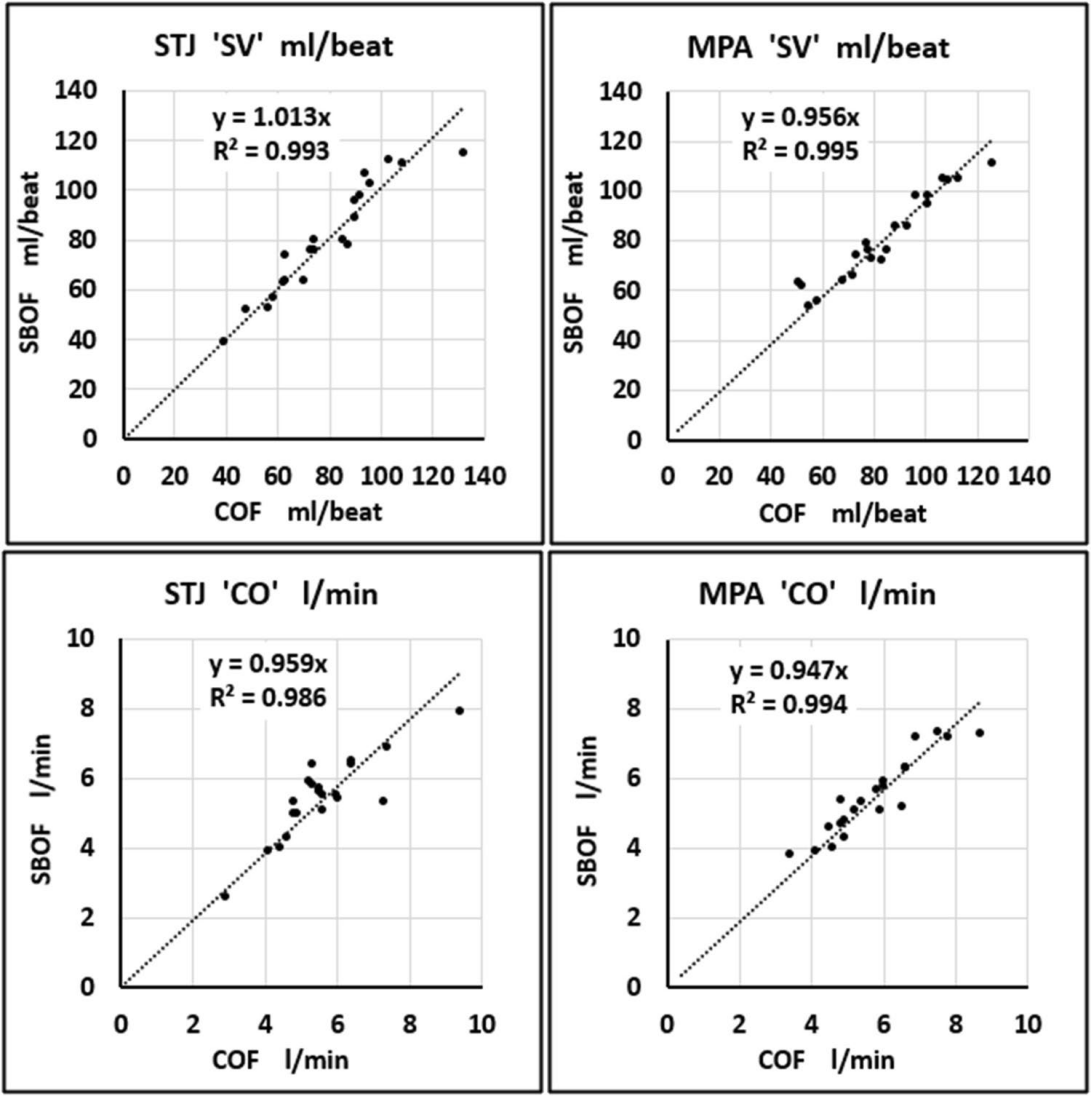
Scatter plots between COF (horizontal) and SBOF (vertical) measurements, for STJ and MPA, in all 21 patients (as ‘SV’ ml/beat and as ‘CO’ l/min). The lower regression slopes (0.956 and 0.947) might reflect the small difference in MPA flow by SBOF compared to COF

Twenty-one patients (Table 2) were enrolled between January 2021 and December 2021 (examples, Figs. 1, 2). The MPA and STJ flows of all 21 patients (plotted in Fig. 3) were included in all of the following results.

**Table 2:** Characteristics of the 21 included patients

Population Characteristics	Mean and range
Age	13.9 y(10–17)
Sex	6 Female; 15 Male
CHDs	11 patients
Non-CHDs	10 patients
Height (m)	1.66 (1.45–1.88)
Weight (kg)	57.6 (36–78)
BSA (m^2^)	1.62 (1.22–1.93)

The BH durations of the phase-contrast flow scans were COF mean 11.7 s (range 8.4–20.9 s) vs SBOF mean 6.5 s (range 3.6–9.1 s). The HRs during the two flow scans were COF mean 71 bpm (range 53–99 bpm) vs SBOF mean 69 bpm (range 55–103 bpm) with paired t-test analysis of mean difference marginally significant (2-tailed for either direction of change in HR) at *p* = 0.054.

**Fig. 4:**
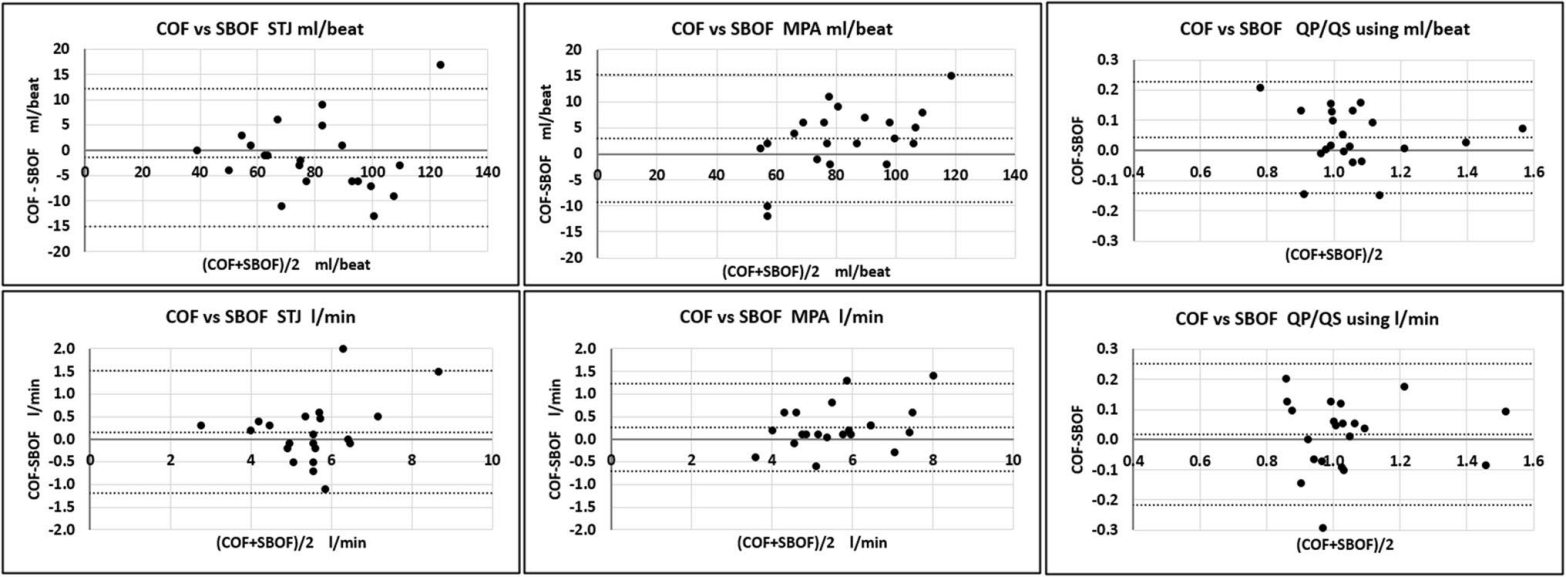
Bland–Altman plots (COF-SBOF difference vs mean of COF and SBOF) of the flow results (as SV and as CO) for both vessels and QP/QS in all 21 patients. For each vessel and QP/QS, and for SV and CO, the mean difference with ± 2SD are the dotted lines

Figure 4 and Table 3 present the overall results of the flow measurements (all *n* = 21 for both vessels and both methods). The 2-tailed paired *t* test *p*-value results (21 pairs) for the mean differences are also included. The STJ showed no significant difference, whereas the MPA showed a significant difference as did weakly the QP/QS by ml/beat.

**Table 3:** Mean differences (COF-SBOF) for STJ, MPA, and Qp/Qs by SV and by CO

	SV (ml/beat) *p*	CO (l/min) *p*
STJ	− 1.43 ± 13.6 0.36	0.16 ± 1.35 0.30
MPA	2.95 ± 12.3 0.04	0.27 ± 0.96 0.02
Qp/Qs	0.04 ± 0.19 0.05	0.02 ± 0.23 0.52

## Discussion

In our study, we acquired COF and SBOF at the CMR studies of 21 paediatric patients referred to the CMR clinical service of the Royal Brompton Hospital, during routine clinical work, as opposed to a potentially more tightly-controlled research setting. Both sequences were acquired with breath holding, and the SBOF typically decreased the flow scan duration to 56% of the COF duration.

For both MPA and STJ, the stroke volumes, considered as the net amount of flow passing through one vessel in a cardiac-cycle, and the cardiac output derived by the multiplication between SV and the HR recorded as an average during the flow acquisition were analyzed.

The SBOF of MPA showed a mean reduction by 3 ml of stroke-volume compared to COF, which nevertheless was detected statistically in these results. There may be genuine changes in flow in the first few cycles of the end-expiratory BH [20, 21], that perhaps are also dependent on how deeply each patient complies with “breathe in, breath out and stop there” end-expiration requests. Possibly a short-term effect was exposed in those first few end-expiratory heartbeats by SBOF as opposed to COF. This result somewhat confounds the overall aim of this paper to improve accuracy by CS shortening of the BH time to assist patients with compliance, but the overall aim may still be valid: namely that the image quality is preserved by the good compliance with the shortened BH of SBOF compared to longer breath-holds needed for COF. The application of a correction factor to adjust SBOF results towards those of COF might be considered.

The 95% CI in these results between COF and SBOF can be viewed in the context of the test–retest 95% CI of COF obtained before this study in a separate paediatric population at our centre (Supplementary 1). The 95% CI between COF and SBOF (LVSV ± 13.6 ml, RVSV ± 12.3 ml, LVCO ± 1.35 l/min, RVCO ± 0.96 l/min, QP/QS by SV ± 0.19, QP/QS by CO ± 0.23) was similar to the previously obtained test–retest 95% CI of COF alone (LVSV ± 10.9 ml, RVSV ± 11.3 ml, LVCO ± 1.2 l/min, RVCO ± 1.1 l/min, QP/QS by SV ± 0.22, QP/QS by CO ± 0.27) (from Supplementary). This suggests that the SBOF method per se is probably not causing any measurable increase in the scatter. The 95% CI found between COF and SBOF, and the 95% CI found in COF might both arise from genuine physiological flow changes, possibly in terms of breath-hold differences and in pre and post-load and heart rate.

Despite the mostly encouraging results, in that no further scatter in flow caused by SBOF compared to COF was detected, our study has some limitations. Firstly, the data have been acquired in a clinical context, and not all the patients were undergoing the same protocol causing potential alteration of the compliance. Secondly, the SBOF scans were collected for ethical reasons a long time after the COF scans, because the entire clinical prescription of scanning had to be completed first, leaving open the risk of patient bulk motion causing misregistration and changed heartrate and BH behaviour between COF and SBOF scans. We note, however, that both of these factors could be seen as encouraging, in that despite all of these potentially deleterious factors, no degradation in 95% CI could be attributed to the use of SBOF because the basic 95% CI of COF alone was similar (The presence of late-washout phase GBCA for SBOF in some patients was assumed to have negligible effect on flow measurements).

Finally, we discuss our work in the light of other studies in the literature that have investigated the use of CS in CMR flow sequences. CS has also been applied to other types of sequences such as cine and 4D flow sequences showing good results in terms of time reduction and cardiac output [24].

Oscanoa et al. [22] investigated the use of Deep learning (DL)-based reconstruction framework to highly accelerated 2D PC flows cine images without losing the accuracy of quantitative measurements. DL-based methods learn the prior image model directly from fully sampled datasets and the major difference from CS is the denoising step, where a convolutional neural network applies a data-driven image model learned directly from previously acquired datasets.

Oscanoa et al. [22] applied their 8 × CD-DL acceleration prospectively in the aorta or main pulmonary artery of 5 pediatric patients, in 24% of their 1.5 × Parallel-Imaging reference scan time) [22], a faster acceleration than by SBOF taking 56% of the COF BH time (approx 1.8 × Parallel-Imaging). The 8 × CD-DL method found RMSE 6.3% for flow measurement (95% CI -11.6, 19.4) (% of ml/beat SV) (*n* = 5). The RMSE of the LV and RV stroke volumes was 8.3% (*n* = 42), as defined in [22] (Table 2 caption). Their 8 × CD-DL prospective method showed similar RMSE performance (6.3%) to SBOF (8.3%), although this statement perhaps depends on the significance of the *n* = 5 result. The 8 × CD-DL gave a 95% CI ≈ ± 15% (% of ml/beat, *n* = 5) which might be compared with ≈ ± 13 ml/beat (n = 42) of SBOF. The median SV by SENSE2 [22] was 88 ml/beat, of which ± 15% is ± 13 ml/beat, again a similar performance with COF-SBOF.

Kocaoglu et al. [23] applied CS to free-breathing flow sequences acquired at the ascending aorta (Ao), descending aorta (DAo), and superior vena cava (SVC) in a transverse plane at the level of pulmonary artery bifurcation levels; the group compared the new sequences with the conventional ones with the intent to find additional acceleration to further shorten acquisition times in patients with complex anatomy. Across 28 patients, they obtained a good correlation with stroke volume, cardiac output and mean peak velocity measurements by standard sequences, evaluating a range of CS accelerations up to 65% reduction in the acquisition time [23]. The free-breathing scanning enabled CS of techniques (to oversimplify, averaging) effective against physiological variations in flow (with respiratory phase but also arrhythmia) that may strongly benefit accuracy, while the CS enables a total scan time that is short enough for routine clinical use where rescans are often required, for example, to optimize VENC or phase-encode FOV aliasing. In our breath-hold work, which used a similar temporal CS acceleration to that of the CS = 4 in Kocaoglu et al. the flow acquisitions were obtained in the oblique planes used in clinical routine. While the planes used are not be expected to cause any difference in the performance of CS, our work including the baseline scatter of the conventional flow method as a reference might be a useful test of CS performance in a routine CMR clinic.

Comparing this work with the Kocaoglu CS4 results, their scan time (free-breathing) was reduced by 45% compared to their SENSE factor 2 reference scan, close to the 44% reduction of BH time in this work by SBOF compared to the COF 1.8 × parallel-imaging scan. Reading the Kocaoglu ascending aorta results for the difference of CS4 from the reference SENSE factor 2 (S2-CS4) [23] (Fig. 3 of Ref 23), middle lower row) the median of the signed difference was 2 ml/beat, with first quartile at − 2 ml/beat and third at + 4 ml/beat, interquartile range 6 ml/beat, so 95% CI ≈ ± 9 ml/beat (n = 28). Compared with the SBOF vs COF results, with similar MD (STJ 1.4 ml/beat), CS4 outperformed the SBOF 95% CI ± 14 ml/beat (STJ, *n* = 21), perhaps because of the free-breathing method.

In conclusion, we demonstrate that the CS application (as SBOF) reduces the breath-hold duration of acquisition to 56% of that of the conventional flow scan (COF). A bias in RV flow by SBOF compared to COF was found. For LV and RV flows, and derived QP/QS, the variation (95% CI) between COF and SBOF was similar to the variation (95% CI) for intrasession test–retest of COF.

### Supplementary Information

Below is the link to the electronic supplementary material.

Supplementary file1 (DOCX 187 KB)

## Data Availability

The DICOM images are all available on a justifiable request to the corresponding author.

## References

[1] Kilner PJ, Geva T, Kaemmerer H, Trindade PT, Schwitter J, Webb GD (2010). Recommendations for cardiovascular magnetic resonance in adults with congenital heart disease from the respective working groups of the European society of cardiology. Eur Heart J.

[2] Valsangiacomo Buechel ER, Grosse-Wortmann L, Fratz S, Eichhorn J (2015). Indications for cardiovascular magnetic resonance in children with congenital and acquired heart disease: an expert consensus paper of the Imaging Working Group of the AEPC and the cardiovascular magnetic resonance section of the EACVI. Eur Heart J Cardiovasc Imaging.

[3] Baumgartner H, De Backer J, Babu-Narayan SV, ESC Scientific Document Group (2021). 2020 ESC Guidelines for the management of adult congenital heart disease. Eur Heart J.

[4] Devos DG, Kilner PJ (2010). Calculations of cardiovascular shunts and regurgitation using magnetic resonance ventricular volume and aortic and pulmonary flow measurements. Eur Radiol.

[5] Lee VS, Spritzer CE, Carroll BA (1997). Flow quantification using fast cine phase-contrast MR imaging, conventional cine phase-contrast MR imaging, and doppler sonography: in vitro and in vivo validation. AJR Am J Roentgenol.

[6] Thunberg P, Karlsson M, Wigström L (2003). Accuracy and reproducibility in phase contrast imaging using SENSE. Magn Reson Med.

[7] Beerbaum P, Körperich H, Gieseke J, Barth P, Peuster M, Meyer H (2005). Blood flow quantification in adults by phase-contrast MRI combined with SENSE—a validation study. J Cardiovasc Magn Reson.

[8] Ferrigno M, Hickey DD, Linér MH, Lundgren CE (1986). Cardiac performance in humans during breath holding. J Appl Physiol.

[9] Nakahira J, Obara S, Jiang Z, Yamaguchi H (1993). Effects of breath holding in air on cardiac responses in man. Jpn J Phys Fitness Sports Med.

[10] Sakuma H, Kawada N, Kubo H (2001). Effect of breath holding on blood flow measurement using fast velocity encoded cine MRI. Magn Reson Med.

[11] Ley S, Fink C, Puderbach M (2006). MRI measurement of the hemodynamics of the pulmonary and systemic arterial circulation: influence of breathing maneuvers. AJR Am J Roentgenol.

[12] Johansson B, Babu-Narayan SV, Kilner PJ (2009). The effects of breath-holding on pulmonary regurgitation measured by cardiovascular magnetic resonance velocity mapping. J Cardiovasc Magn Reson.

[13] Fratz S, Chung T, Greil GF (2013). Guidelines and protocols for cardiovascular magnetic resonance in children and adults with congenital heart disease: SCMR expert consensus group on congenital heart disease. J Cardiovasc Magn Reson.

[14] Kocaoglu M, Pednekar AS, Wang H, Alsaied T, Taylor MD, Rattan MS (2020). Breath-hold and free-breathing quantitative assessment of biventricular volume and function using compressed SENSE: a clinical validation in children and young adults. J Cardiovasc Magn Reson.

[15] Lustig M, Donoho D, Pauly JM (2007). Sparse MRI: The application of compressed sensing for rapid MR imaging. Magn Reson Med.

[16] Hatipoglu S, Gatehouse P, Krupickova S (2022). Reliability of pediatric ventricular function analysis by short-axis "single-cycle-stack-advance" single-shot compressed-sensing cines in minimal breath-hold time. Eur Radiol.

[17] Bland JM, Altman DG (1986). Statistical methods for assessing agree- ment between two methods of clinical measurement. Lancet.

[18] McGill R, Tukey JW, Larsen WA (1978). Variations of box plots. Am Stat.

[19] Wuensch K, Evans J (1996). Straightforward statistics for the behavioral sciences. J Am Stat Assoc.

[20] Thompson RB, McVeigh ER (2006). Cardiorespiratory-resolved magnetic resonance imaging: measuring respiratory modulation of cardiac function. Magn Reson Med.

[21] Feihl F, Broccard AF (2009). Interactions between respiration and systemic hemodynamics Part I: basic concepts. Intensive Care Med.

[22] Oscanoa JA, Middione MJ, Syed AB, Sandino CM, Vasanawala SS, Ennis DB (2022). Accelerated two-dimensional phase-contrast for cardiovascular MRI using deep learning-based reconstruction with complex difference estimation. Magn Reson Med.

[23] Kocaoglu M, Pednekar A, Tkach JA, Taylor MD (2021). Quantitative assessment of velocity and flow using compressed SENSE in children and young adults with adequate acquired temporal resolution. J Cardiovasc Magn Reson.

[24] Pathrose A, Ma L, Berhane H, Scott MB (2021). Highly accelerated aortic 4D flow MRI using compressed sensing: Performance at different acceleration factors in patients with aortic disease. Magn Reson Med.

[25] Walker PG, Cranney GB, Scheidegger MB, Waseleski G, Pohost GM, Yoganathan AP (1993). Semiautomated method for noise reduction and background phase error correction in MR phase velocity data. J Magn Res Img.

