# Supplementary material for: Impact of compressed sensing (CS) acceleration of two-dimensional (2D) flow sequences in clinical paediatric cardiovascular magnetic resonance (CMR)
Source: MAGMA. 2023 May 18;36(6):869–76. doi: 10.1007/s10334-023-01098-8 (PMC10667407; doi:10.1007/s10334-023-01098-8)
Supplement: Supplementary file 1 — Supplementary file1 (DOCX 187 KB) [file 10334_2023_1098_MOESM1_ESM.docx]

**Supplementary: Intrasession Variation of Conventional flow (COF)**

**Background:**

The intrasession variation, let alone full intersession reproducibility, of cine 2D through-slice PC (the conventional breath-hold flow) (COF) measurements in pediatric patients was unknown at our centre and as far as we know has not been published, perhaps because acquisition techniques vary; for example, one major variation is whether breath-hold or free-breathing is applied.

This study therefore assesses the intrasession test-retest variation of the COF method as used in this work in the ascending aorta and main pulmonary artery, keeping acquisition parameters and patient position constant between the repeated COF flow scans.

**Methods:**  51 children and adolescents (mean age: 15 years, range: 9-17 years) with congenital heart disease undergoing routine clinical CMR flow imaging (VE11C SP1, 1.5T, Avanto FIT and Aera, Siemens Healthcare GmbH) were included. Aortic and main pulmonary artery 2D PC flow acquisitions were planned from cine images of the left ventricular outflow tract and the right ventricular outflow tract in two orthogonal planes respectively were acquired. The imaging plane for the 2D PC flow measurements was defined from the acquired cine images. For aortic flow measurements the plane was placed perpendicular to the ascending aorta at the end-systolic level of the sinotubular junction (STJ) in the two left ventricular outflow tract views. Main pulmonary artery flow was measured by using at end-systolic plane just above the pulmonary valve (MPA) in the two right ventricular outflow tract cine images. These views were used for the repeat flow quantification by copying image position. The radiographers selected which of three flow imaging sequence setups (Table S1) to apply depending on the patient’s BH capability (or free breathing (FB)) as per usual practice. Of these three choices, the Long BH setup corresponds to the COF setup in the manuscript. The ShortBH setup in this Supplement did not use CS. CVI42 (5.10; Circle CVI) flow analysis (single observer, 25 years’ experience) used background corrections from static tissue. When repeated SV changed by more than 15ml, the acquisition and analysis were scrutinized for mistakes. After testing for overall differences by paired t-testing, the “95% CI” was defined at ±2SD of the difference between the intrasession test and retest (i.e. ≈1 in 20 exceeds this) for LVSV, RVSV, LVCO, RVCO, QP/QS (by SV and by CO).

**Results**: Figure S1 plots all 51 STJ and 51 MPA flow test and retest results. Figure S2 shows Bland-Altman plots of the 51 STJs and 51 MPAs comparing the test suffixed 1 and retest suffixed 2 in each session, with QP/QS ratios derived from test 1 and from retest 2. The paired t-test results in Table S2 showed that no significant biases occurred between repeat scans in HR, LVSV, RVSV, LVCO, RVCO (p>0.05, n=51, except RVCO p=0.045). The mean differences and 95% CIs as defined in Supplementary Methods above are shown in Table S2. The variability between the repeated flow scans did not appear to depend on the number of heartbeats taken for each flow scan (NHB) (Figure S3) .

**Discussion:** The QP and QS used for QP/QS ratio were usually acquired sequentially to reduce any impact of changing HR. Table S2 shows that the marginally significant test-retest bias for RVCO did not somehow arise from heart-rate changes between the Test (1) and Retest (2) scans, it remains unexplained, and at p=0.05 significance level this may be a Type 1 error. It may be relevant that the Retest (2) scan was usually run ≈ 30 minutes later than the Test (1) scan, after all of the required clinical scans had been completed (as was done in the main COF vs SBOF work).

Flow acquisitions require inevitably inconsistent compromises at larger PE FOV body habitus, or poor BH capacity in the clinical environment; the absence of clearly poorer variation with smaller NHB is unfortunately inconclusive. Further, only two patients were acquired free-breathing and again inconclusive. Post-scanning flow analysis should not degrade variability; however, significant pitfalls exist during both analysis and acquisition. This work was also needed to establish the 95% CI of the usual clinical routine before comparison with fast flow methods (such as compressed sensing). This work was limited to intrasession “test-retest variability”, and is not intersession reproducibility.

**Conclusion:** The intrasession “test-retest” 95% CI found was smaller than of clinical impact in cardiology, although as with any complicated test, outliers arise for various reasons, including physiological variations not technical factors.

| **Definitions and Parameter** | **Long BH(COF)** | **Short BH(noCS)** | **Free-breathing** |
| --- | --- | --- | --- |
| HBs (Heartbeats) | 11-15 | 7-10 | 35-40 |
| Rawdata lines (per HB) | 5 | 6 | 7 |
| TR (ms) @ VENC 120 cm/s | 4.4 | 4.0 | 3.5 |
| Time per acquired cine frames (ms) | 44 | 48 | 49 |
| TE (ms)@ VENC 120 cm/s | 2.3 | 2.2 | 1.9 |
| FOV (mm) (PE FOV adapted to plane) | 360 | 360 | 360 |
| Acquired resln FE(mm) PE(mm)@360 FOV | 1.6 2.0 | 1.6 2.0 | 1.6 2.0 |
| PE rawdata zero-filled fraction | 12.5% | 25% | 25% |
| FA (deg) | 20 | 20 | 20 |
| SLT (mm) | 8 | 8 | 8 |
| ADC (Hz/mm)@360FOV | 297 | 367 | 517 |
| Parallel imaging (GRAPPA) factor | 2 | 2 | 3 |
| Parallel imaging reference lines | 24 (in scan) | 24 (prescan) | 24 (in scan) |
| Long-term averaging vs Respiratory motion | No | No | 5 |

Table S1: The parameters for flow methods used in the intrasession test-retest patients. Note that the long BH corresponds to the COF method in the manuscript. The Short BH flow in this supplement is a different setup that did not use CS.

|  | p | Mean difference (bias) (1-2) | Test-Retest 95% CI |
| --- | --- | --- | --- |
| STJ (ml/beat) | 0.39 | 0.7 ml/beat | -10.2, +11.6 ml/beat |
| STJ (l/min) | 0.16 | 0.12 l/min | -1.1, +1.3 l/min |
| MPA (ml/beat) | 0.15 | -1.2 ml/beat | -12.4, +10.1 ml/beat |
| MPA (l/min) | 0.046 | -0.15 l/min | -1.2, +0.9 l/min |
| QP/QS (ml/beat) | 0.24 | -0.02 | -0.23, +0.20 |
| QP/QS (l/min) | 0.08 | -0.03 | -0.30, +0.24 |
|  |  |  |  |
| HR (STJ 1 vs 2) | 0.76 |  |  |
| HR (MPA 1 vs 2) | 0.15 |  |  |

Table S2: Conventional flow “test-retest” statistical results: The p-value is by 2-tailed paired t-testing assuming equal variances (n=51 pairs). The mean difference and 95% CI limits are as plotted on the Bland-Altman Figure S2. The heartrate (bpm) for the test (1) and retest (2) scans are also analysed for any significant differences.


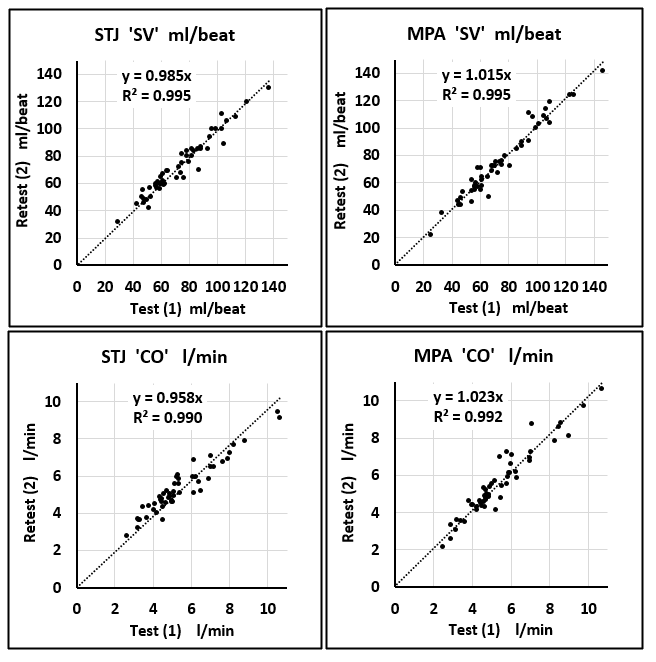


Figure S1: Scatter plots between Test (1) and Retest (2) measurements, for STJ and MPA, in all 51 patients (as ‘SV’ ml/beat and as ‘CO’ l/min).


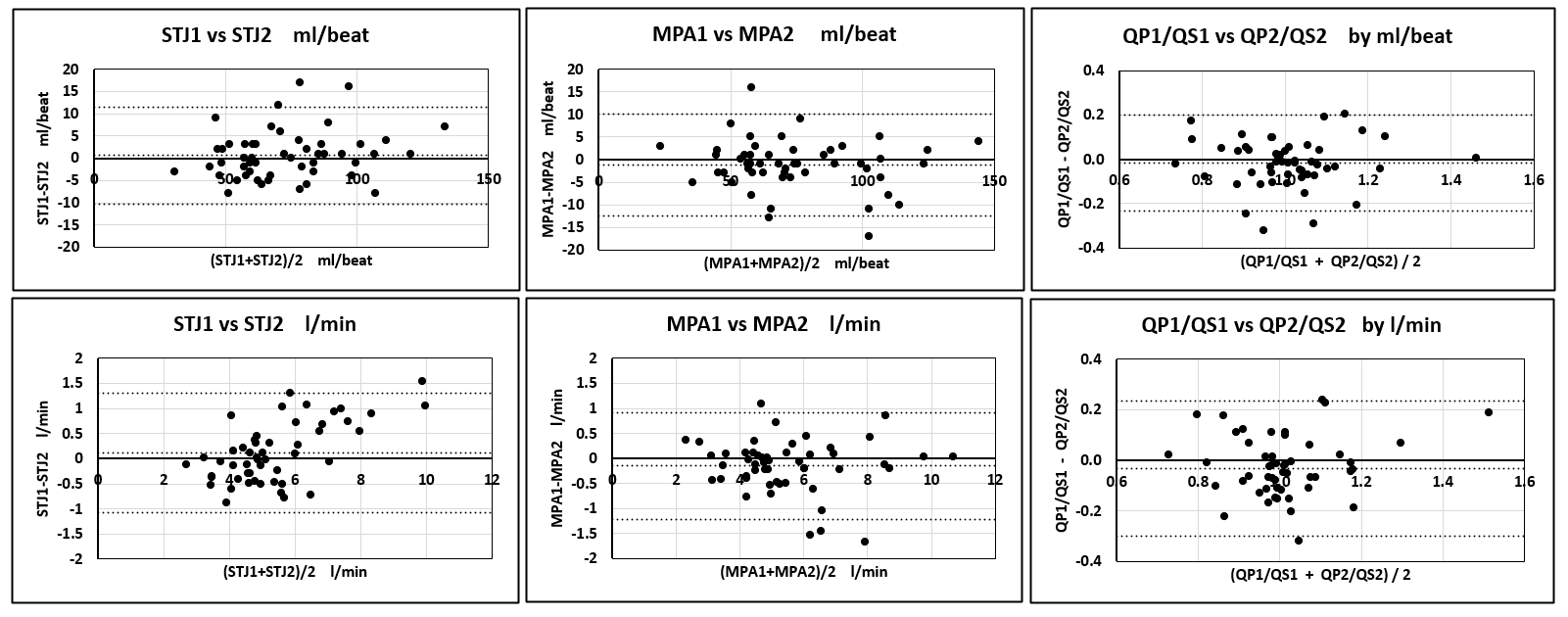


Figure S2: Bland-Altman plots of the test-retest flow scans in 51 patients. For STJ and MPA, the first and second flow scans are identified by suffixes 1 and 2. The QP/QS ratios are derived using QP = MPA flow and QS = STJ flow. All measurements are given twice, in the upper row as the stroke volume in ml/beat, and in the lower row as the cardiac output in l/min (calculated by stroke volume x heartrate (bpm) averaged during the relevant scan). The dotted lines are the mean difference and 95% CI as in Table S2.


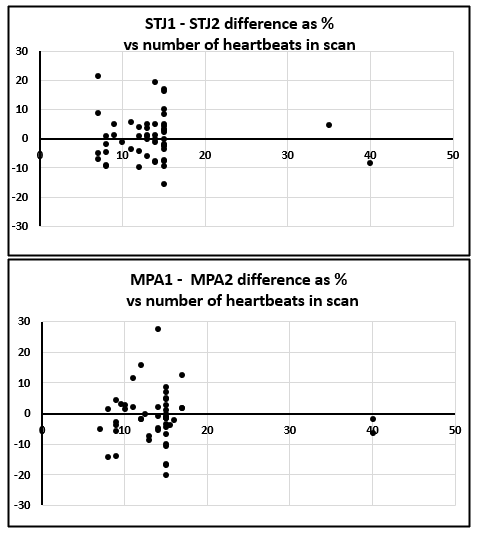


Figure S3: The percentage difference between first and second flow scans as a function of number of heartbeats (NHBs) in each scan. In this case, the difference is expressed as % to remove the association between NHBs in the scan and volume flows, as the number of acquire phase-encoding lines tends to be related to patient size by routine clinical operators. The percentage error does not appear strongly correlated with NHBs. Free-breathing was used for only 2 of the 51 patients.
